# Plasma concentrations of soluble IL-2 receptor α (CD25) are increased in type 1 diabetes and associated with reduced C-peptide levels in young patients

**DOI:** 10.1007/s00125-013-3113-8

**Published:** 2013-11-22

**Authors:** Kate Downes, M. Loredana Marcovecchio, Pamela Clarke, Jason D. Cooper, Ricardo C. Ferreira, Joanna M. M. Howson, Jennifer Jolley, Sarah Nutland, Helen E. Stevens, Neil M. Walker, Chris Wallace, David B. Dunger, John A. Todd

**Affiliations:** 1 JDRF/Wellcome Trust Diabetes and Inflammation Laboratory, Department of Medical Genetics, NIHR Cambridge Biomedical Research Centre, Cambridge Institute for Medical Research, University of Cambridge, Addenbrooke’s Hospital, Cambridge, CB2 0XY UK; 2 Present Address: Department of Haematology, NHS Blood and Transplant, University of Cambridge, Long Road, Cambridge, UK; 3 Department of Paediatrics, University of Cambridge, Addenbrooke’s Hospital, Cambridge, UK; 4 Present Address: Department of Paediatrics, University of Chieti, Chieti, Italy; 5 Present Address: Department of Chemical Engineering and Biotechnology, University of Cambridge, Tennis Court Road, Cambridge, UK; 6 Present Address: Cardiovascular Epidemiology Unit, Department of Public Health and Primary Care, University of Cambridge, Strangeways Research Laboratory, Cambridge, UK; 7 Department of Haematology, NHS Blood and Transplant, University of Cambridge, Long Road, Cambridge, UK

**Keywords:** Autoimmune, Blood, Case–control, CD25, C-peptide, IL-2, IL-2RA Immunoassay, Peripheral, sCD25, Soluble cytokine receptor, Type 1 diabetes

## Abstract

**Aims/hypothesis:**

Type 1 diabetes is a common autoimmune disease that has genetic and environmental determinants. Variations within the *IL2* and *IL2RA* (also known as *CD25*) gene regions are associated with disease risk, and variation in expression or function of these proteins is likely to be causal. We aimed to investigate if circulating concentrations of the soluble form of CD25, sCD25, an established marker of immune activation and inflammation, were increased in individuals with type 1 diabetes and if this was associated with the concentration of C-peptide, a measure of insulin production that reflects the degree of autoimmune destruction of the insulin-producing beta cells.

**Methods:**

We used immunoassays to measure sCD25 and C-peptide in peripheral blood plasma from patient and control samples.

**Results:**

We identified that sCD25 was increased in patients with type 1 diabetes compared with controls and replicated this result in an independent set of 86 adult patient and 80 age-matched control samples (*p* = 1.17 × 10^−3^). In 230 patients under 20 years of age, with median duration-of-disease of 6.1 years, concentrations of sCD25 were negatively associated with C-peptide concentrations (*p* = 4.8 × 10^−3^).

**Conclusions/interpretation:**

The 25% increase in sCD25 in patients, alongside the inverse association between sCD25 and C-peptide, probably reflect the adverse effects of an on-going, actively autoimmune and inflammatory immune system on beta cell function in patients.

**Electronic supplementary material:**

The online version of this article (doi:10.1007/s00125-013-3113-8) contains peer-reviewed but unedited supplementary material, which is available to authorised users.

## Introduction

The IL-2/IL-2 receptor α (IL-2RA) signalling pathway is essential for the regulation of immune responses. Targeted disruption of IL-2 and IL-2RA in mice causes systemic autoimmune disease [1, 2], as do rare *IL2RA* mutations in humans [3, 4]. IL-2RA (CD25) is expressed on many haematopoietic cells, including subsets of T and B cells, most notably regulatory T cells (Tregs), dendritic cells and monocytes, and also non-haematopoietic cells such as endothelial cells, and is upregulated on activation of these cells [5, 6]. The IL-2RA subunit is essential for high-affinity binding of IL-2, and unlike the IL-2RB subunit and the common cytokine receptor γ chain, which bind to other cytokines, the α subunit is unique to IL-2 [6]. IL-2 is largely produced by activated T cells and is required for the generation of functional Tregs [7] and peripheral Treg fitness and maintenance [8, 9].

Upon activation, immune cells proliferate and CD25 is cleaved from the surface by proteases [10–13], including matrix metalloproteinase-2 (MMP-2) and MMP-9 [14–16]. Inhibition of these proteases decreases CD25 cleavage, thus increasing the stability of surface CD25 in vitro [17]. The concentration of sCD25 is age dependent in healthy children, who have high circulating sCD25 concentrations that fall to normal adult concentrations (∼2,000 pg/ml) by age 16–18 years [18]. Elevated sCD25 concentrations in adults are associated with activation of lymphocytes during infection and inflammation, and with autoimmune disease [19–22]. Therefore, sCD25 has been used as a biomarker to help characterise disease progression, prognosis and treatment [23–25]. A previous study of 35 patients with newly diagnosed type 1 diabetes and age-matched controls showed that patients had higher sCD25 concentrations [21]. However, others have reported conflicting results [26, 27].

sCD25 binds to IL-2 in vitro, but with a low affinity (*K*_d_ = 0.03 mol/l) compared with IL-2Rαβγ complex binding of IL-2 (*K*_d_ = 10^−11^ mol/l) [28, 29]. Experiments have shown that, at high concentrations, sCD25 may block IL-2 signalling in vitro [17, 25, 30]. However, at lower concentrations, sCD25 has been shown to potentiate IL-2 signalling [31], as observed with the ligands of other soluble cytokine receptors [32, 33]. Owing to the essential role of IL-2 and the IL-2/IL-2RA pathway for immune homeostasis, the mechanism for cleavage of CD25 from the cell surface and the concentration of sCD25 in the periphery may have an immunoregulatory role and/or indicate immune activation and inflammation.

Here we have measured the concentration of circulating sCD25 in plasma samples from adult (>18 years) patients with type 1 diabetes and healthy adult controls to determine if sCD25 concentrations are associated with disease. Impaired beta cell insulin secretion in patients can be assessed using C-peptide measurements [34]. C-peptide is co-secreted with insulin by the pancreas, as a by-product of the enzymatic cleavage of proinsulin to insulin, and, in patients diagnosed with type 1 diabetes, C-peptide levels decline rapidly because of the autoimmune destruction or inactivation of beta cells. Using C-peptide measurements, we aimed to assess whether there was an association between sCD25 and residual beta cell function in young people with childhood-onset type 1 diabetes and variable diabetes duration.

## Methods

### Samples

For the initial case–control analysis, 200 plasma samples were used from adult patients with type 1 diabetes collected as part of the JDRF/Wellcome Trust GRID cohort (www-gene.cimr.cam.ac.uk/todd/, accessed 1 January 2012). Patients, who were of self-reported white ethnicity, were diagnosed under 17 years of age, and plasma samples were acquired over the age of 18 years. Plasma samples for the 1,600 adult controls were collected as part of the UK Blood Services Common Control Collection (UKBS-CC) [35]. GRID and UKBS-CC samples were collected in acid citrate dextrose solution anticoagulant (Table 1).

**Table 1:** Sample cohorts, sex and age distribution for the case–control experiment, the independent replication case–control experiment and the C-peptide experiment

Experiment	Cohort	Sample number	Proportion of men (%)	Mean age (years)	Age range (years)
Case–control					
Control	UKBS	1,600	52	43.7	18–69
Patient	GRID	200	49	27.6	18–72
Case–control replication					
Control	CBR	80	45	32.5	17–50
Patient	CBR	86	47	33.0	17–50
C-peptide					
Patient	NFS	230	59	14.7	10–20

For the replication study of the case–control analysis, plasma samples from 86 patients and 80 age-matched controls were collected through the Cambridge BioResource (CBR) [5]. All samples for the replication study were collected with the same protocol using EDTA anticoagulant (Table 1).

Non-fasting serum samples for measuring both C-peptide and sCD25 levels were available from 230 young people followed in the Nephropathy Family Study (NFS). The NFS is a prospective study that has, since 2000, recruited more than 1,000 adolescents (10–18 years) with type 1 diabetes and has followed them longitudinally [36]. For the present analysis, 230 patients (age 10–20 years) with variable type 1 diabetes duration, had an available stored serum sample (Table 1). The concentrations of both sCD25 and C-peptide were measured in these samples.

Ethics approval was obtained from the ethics committee, with written consent from participants or parents with assent from the children. All data and samples are treated as confidential. Samples and data are identifiable by a unique barcode only, and volunteers are free to withdraw from these projects at any time. All plasma and serum samples were stored at −80°C.

### sCD25 concentrations

Plasma or serum samples were assayed for sCD25 concentrations using BD OptEIA Human ELISA Kit (BD Biosciences, Franklin Lakes, NJ, USA). The recommended protocol was modified to incorporate mouse IgG, at 10 μg/ml, within the sample diluent.

Europium-labelled streptavidin combined with time-resolved fluorescence spectroscopy was used as the assay readout using DELFIA reagents (Perkin Elmer, Waltham, MA, USA). Each sample was diluted 1:20 in duplicate, and each 100 μl dilution was assayed in the same 96-well plate. Each 96-well plate contained a recombinant sCD25 protein standard curve with a detection range of 31–500 pg/ml. Within the 1,600 UKBS control samples, the mean CV between duplicates was 5.00%.

To assess the reproducibility of the immunoassay, we measured sCD25 concentrations in 40 patient and 40 control plasma samples, in two independent experiments. The two sCD25 concentrations correlated (*r* = 0.86), indicating good reproducibility. In adults, the concentration of sCD25 has been shown to be stable over 12 months [25]. To substantiate this, we measured sCD25 concentrations in 13 adults, with two plasma samples acquired over 6 months apart (mean 236 days). We observed that sCD25 concentrations were stable over this time period (*r* = 0.86). The background level of reactivity, possibly caused by heterophile antibodies, was measured using a mismatched IL-7R detection antibody (human CD127 [IL-7R] biotinylated antibody; eBiosciences, San Diego, CA, USA ) in combination with the standard sCD25 primary antibody. No correlation was observed with sCD25 concentration, and the background concentration was measured using the IL-7R detection antibody in the 40 patient and 40 control plasma samples tested (*r* = 0.03 and *r* = 0.001, respectively).

### C-peptide concentration

C-peptide concentrations were measured using a 1235 AutoDELFIA automatic immunoassay kit from Perkin Elmer . The lower limit of detection was 6.6 pmol/l, and samples with this value were included in analyses and not censored unless described.

Samples were assayed in singleton on a system using a two-step time-resolved fluorimetric assay. All reagents, standards and consumables were those recommended and supplied by the manufacturer. Cross-reactivity with intact proinsulin and 32-33 split proinsulin is ∼60% at 400 pmol/l. Cross-reactivity with intact insulin is <0.1% at 6,000 pmol/l. Between-batch imprecision was 4.0% at 190 pmol/l, 3.8% at 1,125 pmol/l, 1.9% at 277 pmol/l, and 2.9% at 3,898 pmol/l (in-house data).

### Statistical analysis

After graphical examination, sCD25 and C-peptide concentrations were log_10_ transformed to generate a more symmetrical distribution for statistical analysis. To evaluate their relationships with covariates, log_10_ sCD25 or log_10_ C-peptide was used as the dependent variable in multiple linear regressions, with the appropriate covariates as independent variables and Wald tests.

For the initial case–control analysis, sCD25 was measured in GRID type 1 diabetic patients and UKBS control samples, which were randomly split over two ELISA batches. Log_10_ sCD25 concentrations did not differ by batch (*p* = 0.98; type 1 diabetes status was not taken into account because we randomised cases and controls across the two batches). It has been previously shown that sCD25 concentrations are stable in adults [18]. Therefore, we tested this in the 1,600 UKBS control samples and confirmed that sCD25 concentrations in individuals over 18 years of age were not associated with age (*p* = 0.66). As age and batch were not associated with sCD25 concentrations in this dataset, neither was required as covariates in the analysis.

We used regression analysis to compare log-transformed sCD25 concentrations from patient and control samples for the initial analysis of 200 patient and 1,600 control samples, and for the replication analysis of 86 patient and 80 control samples. As the replication samples were age-matched, we also repeated the analysis on a subset of 77 pairs, where both samples had sCD25 and C-peptide concentrations, using a paired *t* test to account for the matching.

For the comparative analysis of sCD25 and C-peptide, up to two serum samples from 230 NFS patients were measured for sCD25 and C-peptide concentrations. Using multiple linear regression analysis, we identified covariates that explained variance in log_10_ sCD25 and log_10_ C-peptide concentrations (age-at-diagnosis and duration-of-disease, respectively; electronic supplementary material [ESM] Table 1, ESM Figs 1 and 2). The linear model we fit assumes a constant rate of change in log_10_ C-peptide concentrations with time since diagnosis. This can only be an approximation to the underlying biological reality, as there must come a time when C-peptide stops decreasing. However, when we attempted to include additional polynomial terms to allow for this, the resulting model predicted that C-peptide levels would start increasing some 7 years after diagnosis. As this is biologically unsound, we chose to use the linear model to adjust log_10_ sCD25 for age-at-diagnosis and log_10_ C-peptide concentrations for duration-of-disease. We then tested whether the log_10_ C-peptide residual (‘adjusted log_10_ C-peptide’) was a significant predictor of the log_10_ sCD25 residual (‘adjusted log_10_ sCD25’) in the 230 samples.

## Results

### Elevated sCD25 concentrations in type 1 diabetes

**Fig. 1:**
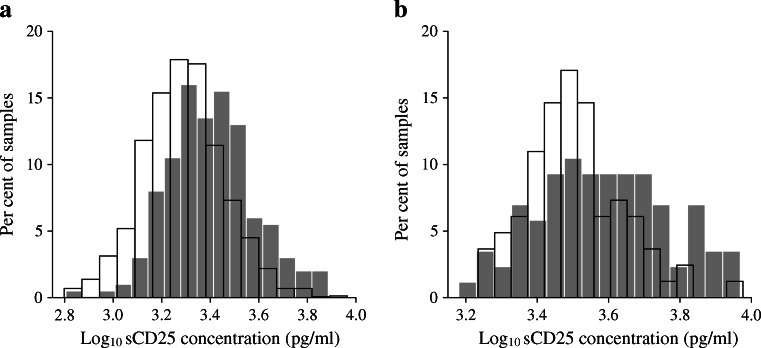
Log_10_ sCD25 concentrations were higher in adult type 1 diabetic patients than in adult control samples. (**a**) 200 type 1 diabetic patients and 1,600 control samples (*p* = 3.12 × 10^−16^) and (**b**) 86 type 1 diabetic patients and 80 control samples (*p* = 1.17 × 10^−3^). Grey bars, type 1 diabetic patients; white bars, controls

We tested for association of sCD25 concentration with type 1 diabetes status using samples from adult patients with type 1 diabetes and controls. Type 1 diabetes was associated with log_10_ sCD25 concentration (*p* = 3.12 × 10^−16^), with concentrations higher in the 200 patient samples than in the 1,600 control samples (Fig. 1a).

Differences in sample collection and/or processing between the patient and control plasma samples could confound this initial observation. Therefore, in order to replicate these findings, we measured sCD25 concentrations in an independent set of 86 type 1 diabetic patients and 80 age-matched adult controls collected and processed using the same protocol. In this replication dataset, log_10_ sCD25 concentration was associated with disease status, with higher concentrations observed in the type 1 diabetic patient samples (mean 4,211 pg/ml) compared with controls (mean 3,356 pg/ml; *p* = 1.17 × 10^−3^; Fig. 1b). This difference was maintained in the subset of 77 matched pairs using a paired *t* test (mean difference 0.091 [95% CI 0.041, 0.140], *t* = 3.67, df = 76, *p* = 4.52 × 10^−4^).

### Increased sCD25 concentrations are associated with decreasing C-peptide concentrations in patients with type 1 diabetes

**Fig. 2:**
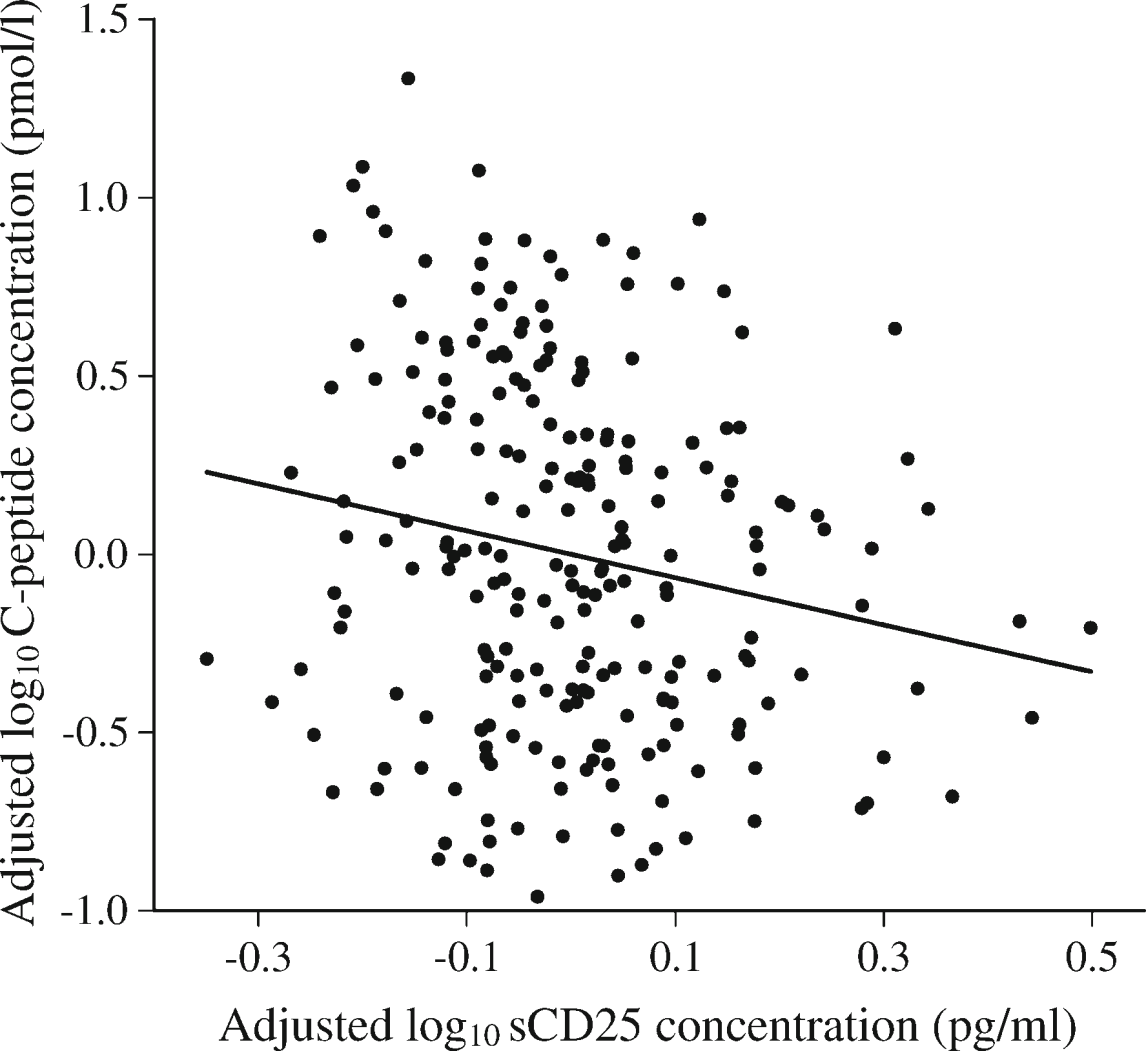
Log_10_ sCD25 and log_10_ C-peptide concentrations were associated in patients with type 1 diabetes under the age of 20 years (*p* = 4.8 × 10^−3^). Residual values are plotted for log_10_ sCD25 and log_10_ C-peptide concentrations that were adjusted for age-at-diagnosis and duration-of-disease, respectively

To determine if sCD25 concentrations were associated with C-peptide concentrations, we analysed measurements from 230 NFS patient samples. The median age was 14.7 years, median duration-of-disease 6.07 years, and 59% of these NFS patients were male. Log_10_ sCD25 concentrations were associated with log_10_ C-peptide concentrations and explained 3.39% of the variance observed (*p* = 4.8 × 10^−3^, regression coefficient −0.051 [95% CI −0.087, −0.02]; Fig. 2). We repeated this analysis, dropping samples at the lower limit of detection for C-peptide concentration (ESM Fig. 2). Within the remaining 181 samples, the association between log_10_ sCD25 concentrations and log_10_ C-peptide concentrations was still observed, with a similar regression coefficient (regression coefficient −0.068 [95% CI −0.117, −0.019], *p* = 6.70 × 10^−3^).

## Discussion

Here we have measured sCD25 concentrations in type 1 diabetic patient and control samples to test if sCD25 concentrations were associated with disease. Circulating sCD25 is used as a biomarker for immune activation [23–25], and previous experiments have also suggested a possible regulatory role for the sCD25 molecule in IL-2 signalling [17, 25, 31].

We know that genetic variation in the *IL2RA* region is associated with susceptibility to type 1 diabetes [37–39], multiple sclerosis [40, 41], Graves’ disease [42], rheumatoid arthritis [43], Crohn’s disease [44], systemic lupus erythematosus [45] and juvenile idiopathic arthritis [46]. Multiple independent association signals within the *IL2RA* region confer risk to type 1 diabetes and are associated with sCD25 concentrations [38, 47, 48]. However, we have observed allelic heterogeneity between the *IL2RA* variants associated with type 1 diabetes and sCD25 concentration [42], and, until much more detailed genetic mapping in larger sample sets is carried out, it will remain unclear if there are causal variants at *IL2RA* shared between sCD25 concentration and risk of type 1 diabetes and other immune diseases.

As sCD25 is associated with age in under 18 year olds, we used adults for our case–control experiments. We identified and replicated an association between sCD25 concentration and disease status, with concentrations 25% higher in adult patients with type 1 diabetes compared with adult control samples (Fig. 1b). This result indicates that sCD25 can be used as a marker for immune activation in patients with, or those at high risk of, type 1 diabetes.

We hypothesised that patients with higher sCD25 concentrations may have more aggressive on-going immune destruction of the pancreatic beta cells and consequently less C-peptide. In samples from 230 patients with type 1 diabetes under 20 years old, we identified that C-peptide concentrations were inversely associated with sCD25 concentrations even in patients with long-duration type 1 diabetes (Fig. 2). These results are based on a small group of young people with type 1 diabetes, and there is a potential limitation related to the measurement of C-peptide, which was performed on non-fasting samples and therefore need to be confirmed in larger studies, including a better assessment of beta cell function. Nevertheless, our findings suggest that sCD25 concentrations could be used as markers for C-peptide loss, and sCD25 could be an informative marker to monitor in immunotherapeutic trials to intervene in the progression of the disease after diagnosis. Our results also add to the large body of literature that indicates a role for immune activation and proinflammatory cytokines, such as TNF-α, IL-1 and IL-6, in the promotion of beta cell death in type 1 diabetes [49]. Rather than sCD25 itself being causal, we think it likely that a raised sCD25 concentration in some patients is a downstream consequence of an active autoimmune inflammatory process.

## Electronic supplementary material

Below is the link to the electronic supplementary material.

ESM Fig. 1 Log10 sCD25 concentration was associated with age-at-diagnosis in the 230 NFS type diabetes patient samples. Variance explained = 5.1%, regression coefficient = -0.0098 (95% CI = -0.015, - 0.005), *p* = 1.96 x 10^-4^(PDF 156 kb)

ESM Fig. 2 Log10 C-peptide concentration was associated with duration-of-disease in the 230 NFS samples. Variance explained = 13.6%, regression coefficient = -0.057 (95% CI = -0.740, - 0.038), *p* = 1.08 x 10^-9^(PDF 158 kb)

ESM Table 1 (PDF 30 kb)

## Abbreviations

CBR: Cambridge BioResource
IL-2RA: IL-2 receptor α
MMP: Matrix metalloproteinase
NFS: Nephropathy Family Study
sCD25: Soluble CD25
Treg: Regulatory T cell
UKBS-CC: UK Blood Services Common Control Collection
