# Supplementary figures and images for: Plasma concentrations of soluble IL-2 receptor α (CD25) are increased in type 1 diabetes and associated with reduced C-peptide levels in young patients
Source: Diabetologia. 2013 Nov 22;57(2):366–72. doi: 10.1007/s00125-013-3113-8 (PMC3890035; doi:10.1007/s00125-013-3113-8)

Electronic Supplementary Material – Figure 1

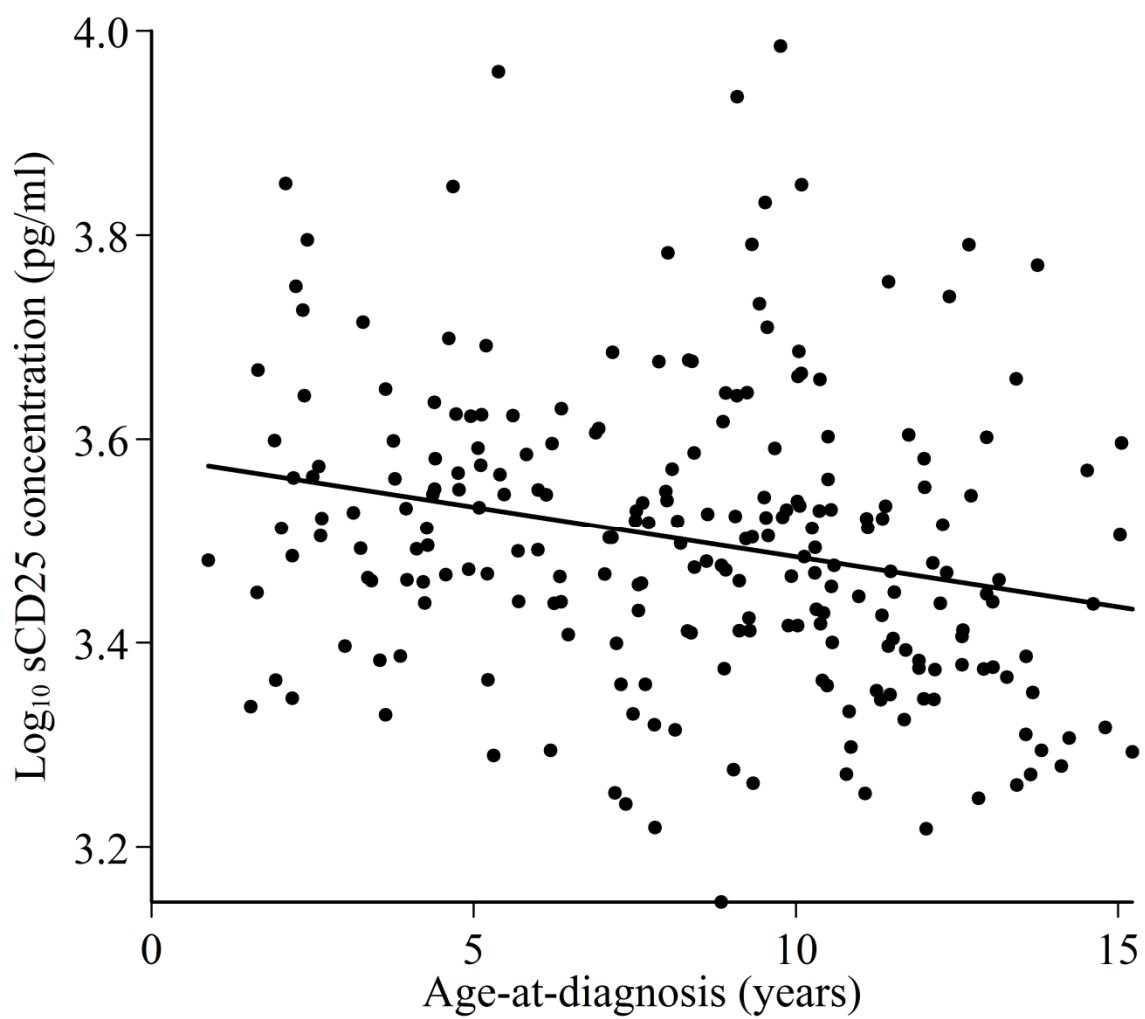

Supplement: Supplementary file 1 — Log10 sCD25 concentration was associated with age-at-diagnosis in the 230 NFS type diabetes patient samples. Variance explained = 5.1%, regression coefficient = -0.0098 (95% CI = -0.015, - 0.005), p = 1.96 x 10^-4^(PDF 156 kb) [file 125_2013_3113_MOESM1_ESM.pdf]

Electronic Supplementary Material – Figure 2

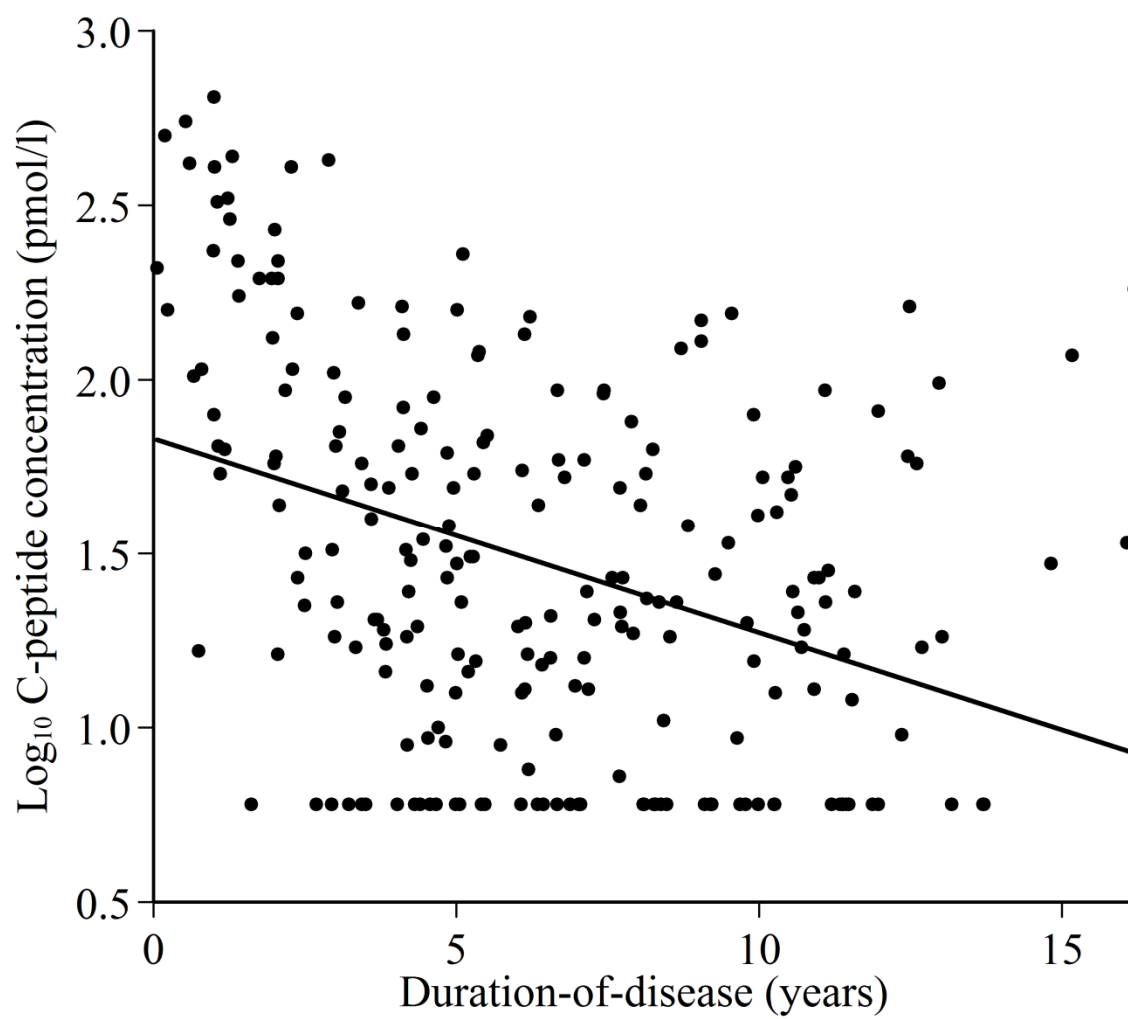

Supplement: Supplementary file 2 — Log10 C-peptide concentration was associated with duration-of-disease in the 230 NFS samples. Variance explained = 13.6%, regression coefficient = -0.057 (95% CI = -0.740, - 0.038), p = 1.08 x 10^-9^(PDF 158 kb) [file 125_2013_3113_MOESM2_ESM.pdf]
