# Supplementary material for: Plasma concentrations of soluble IL-2 receptor α (CD25) are increased in type 1 diabetes and associated with reduced C-peptide levels in young patients
Source: Diabetologia. 2013 Nov 22;57(2):366–72. doi: 10.1007/s00125-013-3113-8 (PMC3890035; doi:10.1007/s00125-013-3113-8)
Supplement: Supplementary file 3 — (PDF 30 kb) [file 125_2013_3113_MOESM3_ESM.pdf]

Supplementary Table 1

Results of covariate analysis of sCD25 and C-peptide concentrations in 230 type 1 diabetes patient NFS samples.

	Covariate	*p*	Coefficient	95% CI
sCD25	Age-at-diagnosis	4.0×10^{-3}	-0.012	-0.021, -0.004
	Duration-of-disease	0.46	-0.003	-0.011, 0.005
	Sex	0.78	-0.005	-0.043, 0.032
C-peptide	Duration-of-disease	3.0×10^{-3}	-0.044	-0.073, -0.015
	Age-at-diagnosis	0.29	0.016	-0.014, 0.045
	Sex	0.51	0.044	-0.089, 0.178
